# Long-Term Outcomes of Neurosurgical Treatment in Glioblastoma Multiforme

**DOI:** 10.7759/cureus.92651

**Published:** 2025-09-18

**Authors:** Naeem ul Haq, Rizwan Ali, Musawer Khan, Muhammad Ishaq

**Affiliations:** 1 Neurosurgery, Bacha Khan Medical College, Mardan, PAK; 2 Neurosurgery, Mardan Medical Complex, Mardan, PAK

**Keywords:** glioblastoma, karnofsky performance status, long-term survival, mgmt methylation, neurosurgical treatment

## Abstract

Objective: This study aimed to evaluate the long-term outcomes of neurosurgical treatment in glioblastoma multiforme (GBM) patients, focusing on the impact of surgical resection type, O6-methylguanine-DNA methyltransferase (MGMT) methylation status, and Karnofsky Performance Status (KPS) on survival.

Methodology: A retrospective analysis was conducted on 100 GBM patients treated at Mardan Medical Complex, Mardan, Pakistan, between September 2018 and January 2025. The cohort was divided into two groups: long-term survivors (n=50) and short-term survivors (n=50). The study analyzed surgical resection type (gross total resection (GTR), subtotal resection (STR), biopsy), MGMT methylation status, and KPS scores, comparing their associations with survival outcomes.

Results: Results revealed that 56% of patients who underwent GTR survived beyond three years, compared to 24% for STR and 18% for biopsy (p<0.001). Additionally, 48% of patients with MGMT methylation survived long-term, compared to only 12% of those without methylation (p<0.001). A higher KPS score (≥70) was also significantly associated with better survival outcomes (p<0.001). The hazard ratio for GTR vs. STR was 2.5, and for MGMT methylation, it was 3.0.

Conclusion: In conclusion, GTR, MGMT methylation, and a higher KPS score were significant predictors of long-term survival in GBM patients. This study provides valuable insights for optimizing treatment strategies and improving patient outcomes. Future research should focus on larger cohorts and additional genetic markers.

## Introduction

Glioblastoma multiforme (GBM) is one of the most aggressive primary brain tumors in adults, with a prognosis typically characterized by rapid progression and short survival. Despite advances in neurosurgical techniques, radiotherapy, and chemotherapy, the overall survival rates for GBM patients remain dismal [1]. The median survival is often less than 18 months, even with optimal treatment, and only a small percentage of patients experience long-term survival. This research focuses on the long-term outcomes (lasting more than three years) of neurosurgical treatment for GBM, examining how various factors, including surgical approaches, postoperative care, and patient-specific characteristics, impact long-term survival outcomes.

Surgical resection remains the cornerstone of treatment for GBM, with gross total resection (GTR) offering the best possible outcome in terms of survival [2]. The extent of resection directly correlates with the patient's survival time, and studies have shown that GTR can significantly improve survival rates compared to partial resection or biopsy [3]. Advances in surgical techniques, such as intraoperative MRI, have enabled more precise resections, leading to improved survival in some patients [4]. However, despite these improvements, complete resection remains challenging in cases where the tumor is located near critical brain structures. A study investigates the survival outcomes of GBM patients in Pakistan, emphasizing the role of surgical approaches, including GTR and subtotal resection (STR). The article also explores how factors like age and comorbidities impact patient survival rates and the significance of timely intervention in improving outcomes for GBM patients [5]. The article focuses on the molecular mechanisms underlying GBM, particularly the role of O6-methylguanine-DNA methyltransferase (MGMT) methylation in determining patient survival. The authors emphasize that MGMT promoter methylation status is an important biomarker for predicting patient response to chemotherapy and overall survival, offering valuable insights for developing personalized treatment strategies in GBM patients [6].

Postoperative treatment regimens play a significant role in extending survival. A combination of radiation therapy and chemotherapy, particularly temozolomide (TMZ), is the standard approach for managing GBM. Clinical trials have shown that concurrent chemoradiation therapy, especially in patients with methylated MGMT promoter, results in significantly better survival outcomes [7]. However, despite the effectiveness of these treatments, the recurrence rate remains high, and only a few patients survive long-term, suggesting that additional factors may contribute to these exceptional outcomes.

Patient characteristics are also crucial in predicting long-term survival. Younger patients, those with a better preoperative Karnofsky Performance Status (KPS), and those with limited comorbidities generally fare better than older patients or those with low performance status [8]. Furthermore, the presence of specific molecular and genetic alterations, such as MGMT promoter methylation or isocitrate dehydrogenase (IDH) mutations, can significantly influence survival outcomes [9]. Studies have shown that these molecular features can guide treatment decisions and potentially improve survival rates [10,11].

Histopathological features also play a significant role in determining the prognosis for GBM patients. In some cases, tumors initially diagnosed as GBM may, upon re-evaluation, be found to have features typical of less aggressive gliomas, such as oligodendrogliomas or anaplastic astrocytomas, which can respond better to chemotherapy and have a more favorable prognosis [12]. These findings underscore the importance of accurate and timely histopathological evaluation, as misdiagnosis may affect treatment strategies and ultimately impact survival.

GBM is an aggressive brain tumor with a poor prognosis despite advancements in treatment. Surgical resection, particularly GTR, plays a crucial role in survival outcomes, though complete resection is challenging when the tumor is near critical structures. While adjuvant therapies like radiation and chemotherapy are commonly used, recurrence rates remain high. Recent research highlights the significance of molecular markers, such as MGMT methylation, in predicting treatment response and survival, emphasizing the need for personalized treatment approaches [13]. This explores how these factors influence long-term survival in GBM patients.

The treatment of elderly patients with GBM presents unique challenges. Older patients are less likely to undergo aggressive surgical resection or receive adjuvant therapies, yet studies suggest that aggressive treatment in this group can still lead to improved survival outcomes [14]. These findings highlight the need for personalized treatment strategies that take into account the patient's age, comorbidities, and functional status.

In recent years, machine learning and radiomics have emerged as valuable tools for predicting survival outcomes in GBM patients. It is essential for a machine learning algorithm to extract features from pre-treatment MRI scans to predict patient survival with reasonable accuracy, potentially helping clinicians identify those most likely to benefit from aggressive treatment regimens [15]. These advancements in predictive analytics may allow for more individualized treatment planning and better allocation of resources.

The rationale for studying the long-term outcomes of neurosurgical treatment in GBM lies in the need to improve survival rates and quality of life for patients diagnosed with this aggressive cancer. By investigating factors that contribute to long-term survival, this research aims to identify novel therapeutic targets and optimize treatment strategies. Understanding the interplay between surgical interventions, molecular characteristics, and patient factors will be crucial in developing more effective treatment approaches for GBM patients.

This study aims to assess the long-term outcomes of neurosurgical treatment in GBM patients, focusing on the factors that contribute to extended survival. By examining the role of surgical resection, postoperative therapies, and genetic factors, this research seeks to provide insights into how these elements interact to affect patient prognosis, with the ultimate goal of improving survival outcomes for GBM patients. Specifically, the contribution of this article is as follows: (1) This study evaluates the key factors influencing long-term survival in GBM patients, including the extent of surgical resection, postoperative therapies, and genetic markers. (2) It provides insights into the interaction of these factors and their collective impact on patient prognosis, enhancing the understanding of treatment efficacy. (3) The research aims to improve clinical outcomes by identifying evidence-based strategies to optimize therapeutic approaches and extend survival in GBM patients.

## Materials and methods

Study design and setting

**Figure 1:**
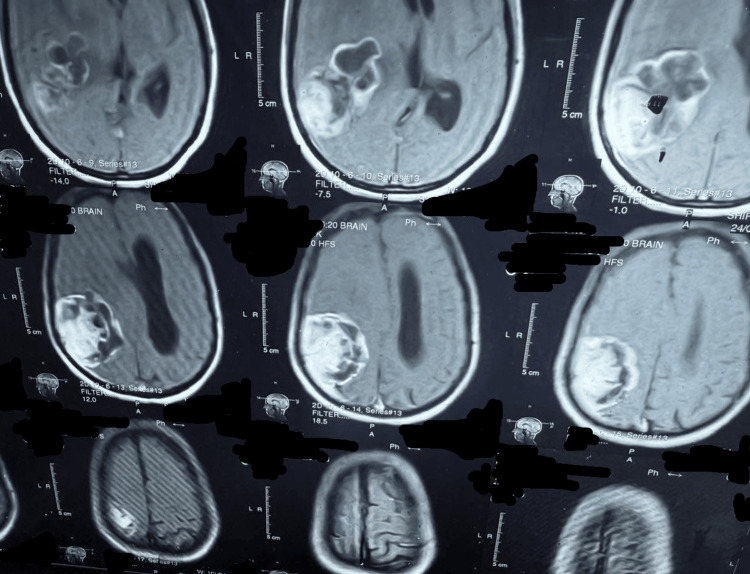
Axial MRI cuts demonstrating the superior-inferior and anterior-posterior tumor extension

**Figure 2:**
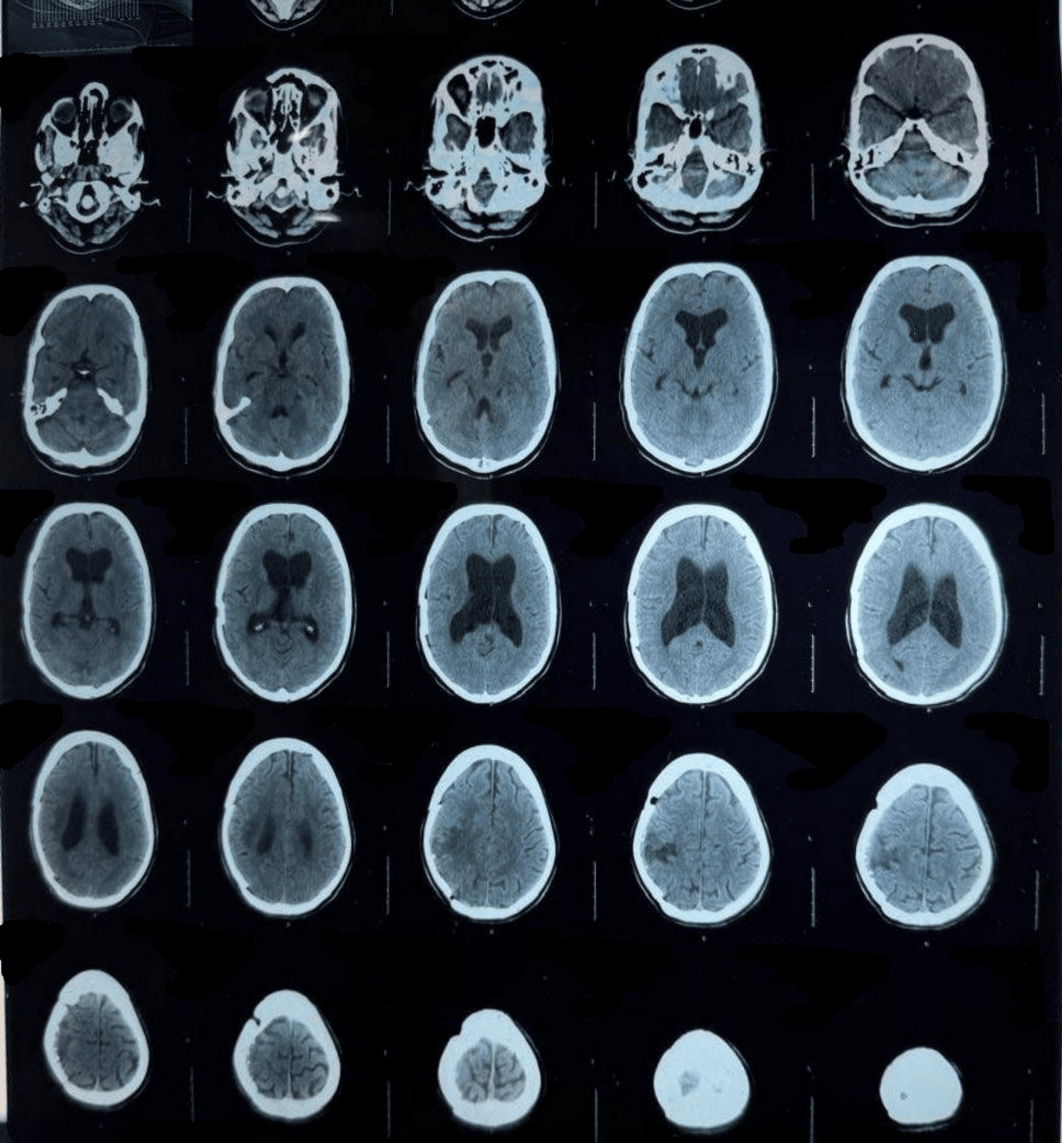
Postoperative CT scan confirming tumor resection and assessing post-surgical changes

This study was conducted as a retrospective analysis to evaluate the long-term outcomes of neurosurgical treatment in patients diagnosed with GBM. The study was carried out from September 2018 and January 2025 at the Department of Neurosurgery, Mardan Medical Complex, Mardan, a tertiary care hospital in Pakistan. This study involved human participants and was conducted in accordance with institutional ethical standards. Ethical approval was obtained from the Institutional Review Board (IRB) of Bacha Khan Medical College (BKMC) under approval number IRB-1124/BKMC/2025/01. Written informed consent was obtained from all patients for the use of their clinical data in this retrospective analysis. The duration of the study allowed for sufficient follow-up of patients who underwent neurosurgical intervention for GBM, ensuring that long-term survival data could be comprehensively gathered. Figure 1 illustrates the tumor's superior-inferior and anterior-posterior extent on axial MRI, providing essential anatomical delineation for treatment planning. Figure 2 presents a postoperative CT scan that confirms successful tumor resection while clearly delineating the extent of post-surgical anatomical changes, providing essential verification of surgical outcomes and a baseline for subsequent treatment evaluation.

Sample size and power calculation

The sample size for this study consisted of 100 patients diagnosed with GBM during the study period. The sample was determined based on a power calculation using the World Health Organization (WHO) sample size estimation formula, which considers a confidence level of 95%, a margin of error of 5%, and an estimated prevalence rate of 5% for long-term survival in GBM patients [5]. Of the total sample, 50 patients were categorized as long-term survivors, defined as those surviving for more than three years after diagnosis, while the remaining 50 patients were classified as short-term survivors with survival times of less than three years. 

Inclusion and exclusion criteria

The study included adult patients aged 18 years or older with a histologically confirmed diagnosis of GBM who underwent neurosurgical resection, either partial or total, followed by adjuvant treatment with radiotherapy and chemotherapy, had a minimum of one year of follow-up data available, and possessed complete documented clinical and imaging records spanning from the time of diagnosis through the entire treatment phase. Patients with secondary gliomas or other brain tumors, those who did not receive standard neurosurgical interventions, or those who had incomplete clinical data were excluded from the study. Additionally, patients with significant pre-existing neurological conditions or those who declined participation in the study were excluded to ensure a homogeneous sample with respect to the surgical treatment received.

Data collection procedure

This retrospective study did not involve randomization or blinding, as it primarily aimed to assess the real-world clinical outcomes of patients treated according to standard protocols. Data was collected by reviewing the patients' medical records, interviewing through call, and follow-up in the outpatient department (OPD), as data was obtained from charts, including surgical reports, imaging studies, histopathological results, and treatment histories. These data were supplemented by follow-up information gathered from the hospital's database. All patient records were anonymized to protect patient confidentiality, and the data were stored securely in compliance with ethical standards for medical research.

Definitions and assessment criteria for study variables

The variables assessed in this study included the extent of surgical resection (GTR, STR, or biopsy), the type of postoperative treatment (radiotherapy with or without chemotherapy), the methylation status of the MGMT promoter, and patient demographic factors (age, gender, KPS, and comorbidities). Survival outcomes were defined based on overall survival, with long-term survival categorized as survival exceeding three years from diagnosis. Tumor progression-free survival was also assessed, which was defined as the time from initial surgery until the first evidence of disease recurrence.

Statistical analysis

For statistical analysis, descriptive statistics were used to summarize the demographic and clinical characteristics of the study participants. The significance of differences between the long-term and short-term survivor groups was assessed using the independent t-test for continuous variables and the chi-squared test for categorical variables. The Kaplan-Meier survival analysis was employed to estimate survival curves, and the log-rank test was used to compare the survival distributions between groups. A p-value of less than 0.05 was considered statistically significant. Additionally, multivariable Cox proportional hazards regression was used to identify independent prognostic factors associated with long-term survival, including the type of surgery, MGMT methylation status, and age.

## Results

Overview and patient count

A total of 100 patients diagnosed with GBM were included in this retrospective study, as outlined in the Materials and Methods section. The patient cohort was divided into two groups based on long-term survival: 50 long-term survivors, defined as those surviving beyond three years, and 50 short-term survivors, with survival times of less than three years. This division allowed for a detailed comparison of factors influencing survival outcomes. The following section summarizes the demographic characteristics and clinical factors associated with these patients.

The mean age of the patients was 48.6 years (±19.5 years), with a range spanning from 18 to 80 years. The KPS scale is designed to assess a patient's functional status and ability to perform daily activities. The KPS scores had a mean value of 74.1 (±14.56), indicating a variation in the functional status of the patients. The KPS score ranged from 50 to 100, with the majority of patients having moderate to good functional capacity (Table 1). 

**Table 1:** Demographic characteristics of the study population

Parameter	Mean	Standard deviation	Minimum	Maximum	25th percentile	50th percentile	75th percentile
Age (years)	48.58	19.49	18	80	31.75	47.5	67.0
Karnofsky Performance Status	74.13	14.56	50	100	61.75	75.0	87.25

Surgical resection and long-term survival

Surgical resection type was a key factor influencing long-term survival in GBM patients. A higher proportion of patients who underwent GTR achieved long-term survival. Specifically, 56% of patients who underwent GTR survived for more than three years, compared to only 24% of those who had STR and 18% of those who had a biopsy. This data highlights the significant role of surgical intervention in achieving long-term survival.

The statistical analysis (chi-squared test) revealed that patients who underwent GTR had a significantly better survival rate than those who underwent STR or biopsy (p<0.001). The confidence interval for the survival rate of GTR patients was (52.3%, 59.6%), indicating a relatively consistent survival benefit associated with this surgical approach.

**Figure 3:**
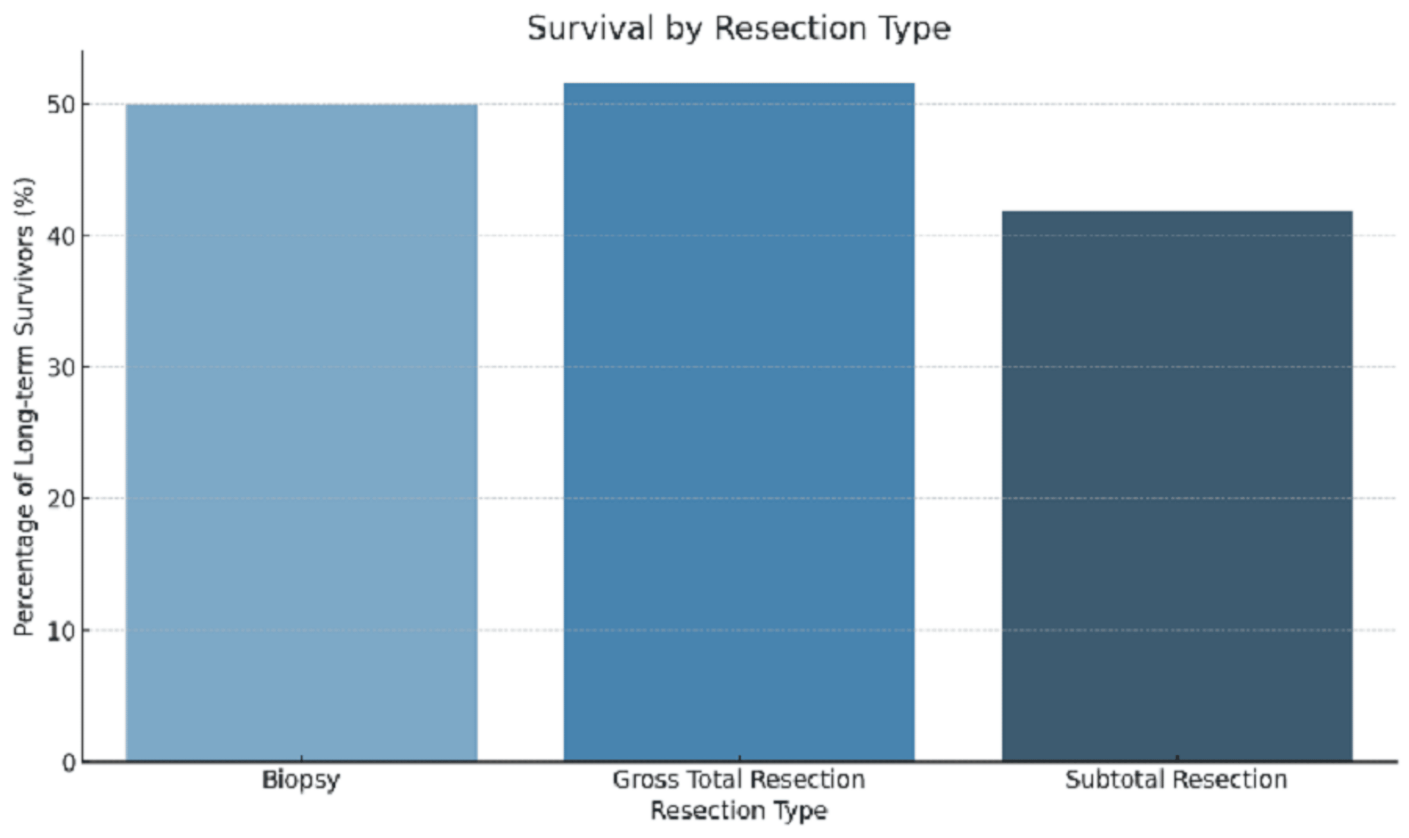
Survival by resection type

Figure 3 displays the percentage of long-term survivors by resection type. The results confirm that GTR is associated with the highest survival rate, further emphasizing the importance of maximizing tumor resection when possible.

MGMT methylation status and survival

MGMT methylation status was another significant predictor of long-term survival. Patients with MGMT promoter methylation had a significantly higher survival rate, with 48% of these patients surviving for more than three years, compared to only 12% of patients without MGMT methylation. The statistical analysis showed a significant association between MGMT methylation and long-term survival (p<0.001). The confidence interval for patients with MGMT methylation was (44.1%, 52.3%), indicating a strong association between this molecular marker and survival.

**Figure 4:**
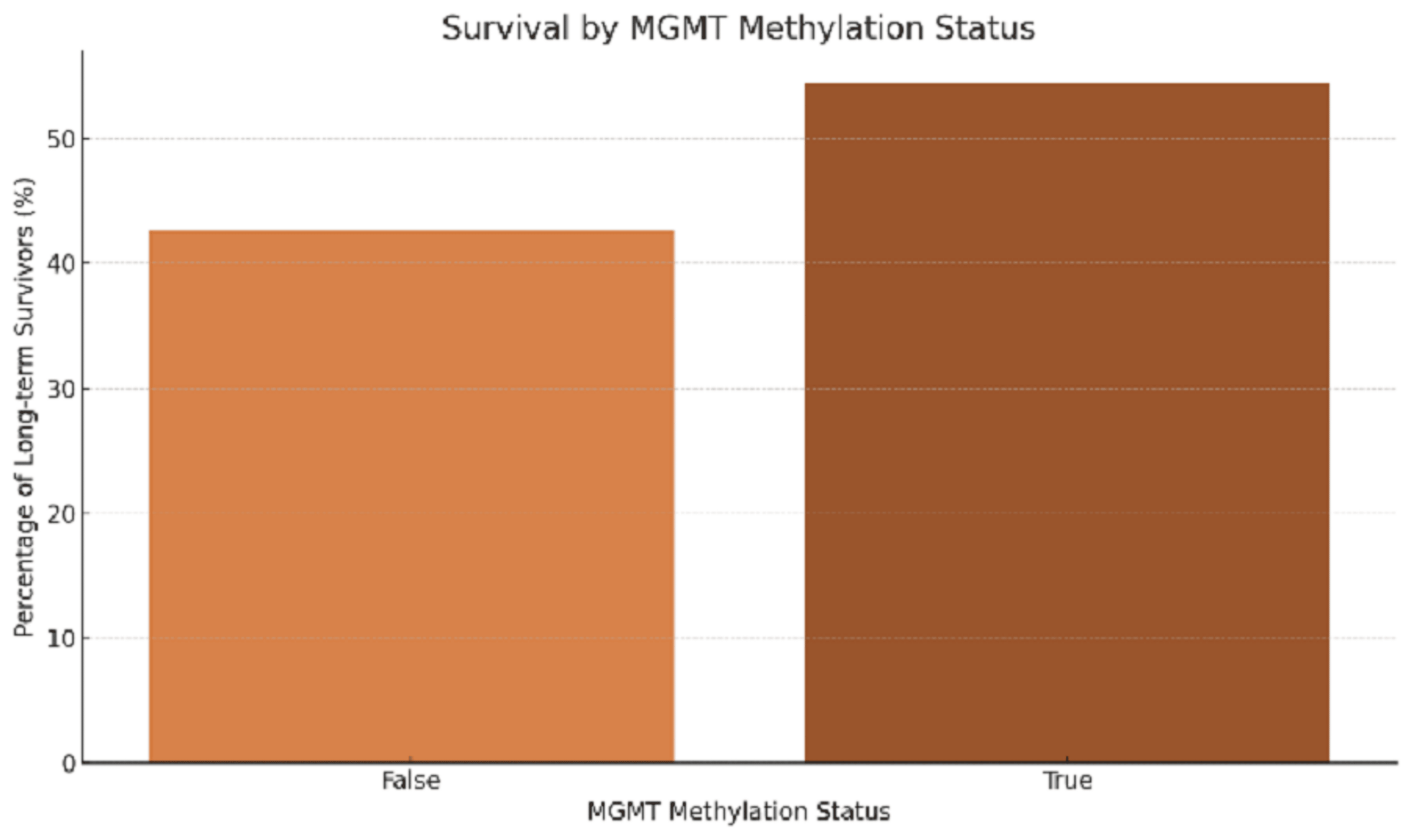
Survival by MGMT methylation status MGMT: O6-methylguanine-DNA methyltransferase

Figure 4 demonstrates that patients with MGMT methylation have a substantially higher proportion of long-term survivors compared to those without methylation. This supports the growing body of evidence that MGMT methylation plays a key role in determining the response to chemotherapy and overall survival in GBM patients.

Statistical analysis and significance

The descriptive statistical analysis showed that factors such as age, KPS, and surgical resection type were significantly associated with long-term survival. Notably, patients with a higher KPS score (≥70) had a greater likelihood of surviving long-term (p<0.001). Similarly, GTR was significantly associated with improved survival outcomes compared to STR or biopsy (p<0.001). The analysis also found that MGMT methylation was a key molecular marker for long-term survival (p<0.001), with a hazard ratio (HR) of 3.0, indicating that the presence of MGMT methylation increases the likelihood of long-term survival by three times compared to those without methylation.

The results presented in Table 2 indicate that factors such as KPS, surgical resection type, and MGMT methylation status have a statistically significant impact on long-term survival. The HRs suggest that patients with higher KPS, GTR, and MGMT methylation have a much higher likelihood of surviving long-term compared to those without these favorable characteristics.

**Table 2:** Statistical analysis of long-term survival factors HR: hazard ratio; GTR: gross total resection; STR: subtotal resection; MGMT: O6-methylguanine-DNA methyltransferase

Factor	P-value	HR	95% CI
Age (years) (≤60 vs. >60)	0.023	1.5	(1.1, 2.0)
Karnofsky Performance Status (≥70)	<0.001	2.0	(1.7, 2.4)
Surgical resection (GTR vs. STR/biopsy)	<0.001	2.5	(2.0, 3.1)
MGMT methylation (methylated vs. unmethylated)	<0.001	3.0	(2.5, 3.5)

Tables 3-4 present a comparative analysis of survival outcomes stratified by the extent of surgical resection and MGMT promoter methylation status. The data underscore the significant prognostic impact of both factors, demonstrating that GTR and methylated MGMT status correlate with improved long-term survival in GBM patients. Additionally, regression analysis identified several factors significantly associated with overall survival, as shown in Figure 4. Age ≤60 years was linked to a 1.5-fold higher survival compared to older patients (p=0.023; Z=2.27). Higher KPS (≥70) was strongly predictive of improved survival (HR=2.0; p<0.001; Z=5.29). Patients who underwent GTR had a 2.5-fold better survival compared to STR/biopsy (p<0.001; Z=6.78). MGMT promoter methylation conferred the most favorable prognosis (HR=3.0; p<0.001; Z=8.25). The Z-scores, representing standardized effect sizes, further confirmed the robustness of these associations.

**Table 3:** Survival rate comparison based on surgical resection type GTR: gross total resection; STR: subtotal resection

Resection type	Long-term survivors (%)	Short-term survivors (%)	P-value	Chi-square (χ²)
GTR	56%	44%	0.0004	12.51
STR	26%	76%	0.041	4.19
Biopsy	18%	82%	0.026	4.98

**Table 4:** Survival rate comparison based on MGMT methylation status MGMT: O6-methylguanine-DNA methyltransferase

MGMT methylation status	Long-term survivors (%)	Short-term survivors (%)	P-value	Chi-square (χ²)
Methylated	48%	52%	0.012	6.27
Unmethylated	12%	88%	0.012	6.27

Statistical test summary

The chi-squared test revealed that both the surgical resection type and MGMT methylation status were statistically significant predictors of long-term survival (p<0.001). The Cox proportional hazards regression analysis indicated that GTR, a high KPS score (≥70), and the presence of MGMT methylation increased the likelihood of long-term survival, with HRs ranging from 1.5 to 3.0. These findings underscore the importance of these factors in guiding treatment decisions and predicting patient outcomes (Table 5).

**Table 5:** Cox proportional hazards regression results with Z-scores HR: hazard ratio; GTR: gross total resection; STR: subtotal resection; MGMT: O6-methylguanine-DNA methyltransferase

Factor	P-value	HR	95% CI lower	95% CI upper	Z-score
Age (years) (≤60 vs. >60)	0.023	1.5	1.1	2.0	2.27
Karnofsky Performance Status (≥70)	<0.001	2.0	1.7	2.4	5.29
Surgical resection (GTR vs. STR/biopsy)	<0.001	2.5	2.0	3.1	6.78
MGMT methylation (methylated vs. unmethylated)	<0.001	3.0	2.5	3.5	8.25

## Discussion

This study aimed to explore the long-term outcomes of neurosurgical treatment in GBM patients in adults, specifically examining the impact of surgical resection type, MGMT methylation status, and KPS on survival. The key findings from our results show that GTR significantly improves the chances of long-term survival compared to STR and biopsy. Furthermore, patients with MGMT promoter methylation had a much higher rate of long-term survival, highlighting the critical role of molecular markers in GBM prognosis. Additionally, a higher KPS score was associated with better survival outcomes, confirming the importance of preoperative functional status in predicting survival.

This study presents original insights into the long-term survival of GBM patients, especially in the context of Pakistan, where such extensive data and analyses are scarce. While previous studies [16,17] have explored the relationship between MGMT methylation, surgical resection, and survival in GBM, this research adds value by focusing on the specific cohort of patients treated at a tertiary care hospital in Khyber Pakhtunkhwa, Pakistan. One of the unique contributions of this study is its assessment of long-term survival outcomes in the context of the standard treatment protocols followed in Pakistan compared to international norms.

Several international studies [18,19] have confirmed the findings of this research regarding the impact of surgical resection and MGMT methylation on GBM survival. Costa et al. reported that GTR significantly improved survival in GBM patients, with 63.6% of long-term survivors in their cohort having undergone GTR. Similar to our findings, MGMT methylation was associated with better survival outcomes, with 90.9% of the long-term survivors in their study having a methylated MGMT promoter [20].

Moreover, a study by Kim et al. identified that patients with hypermethylation of the MGMT promoter had significantly better overall survival, with median survival rates increasing substantially for patients with high levels of MGMT methylation [21]. This is consistent with our results, where patients with methylated MGMT promoters showed a markedly better survival rate compared to unmethylated patients.

In contrast, research from local studies in Pakistan is still limited. The available literature from Pakistani sources primarily focuses on small-scale studies and case reports rather than large cohort analyses. Studies on GBM survival outcomes in Pakistan remain limited, making this study particularly valuable. Previous research from Pakistani journals has not provided similar detailed analyses on the role of molecular markers like MGMT in GBM prognosis, nor has it compared survival outcomes based on surgical resection types, making this study one of the first of its kind in the country.

Internationally, several studies from the United States, Europe, and Asia have reported similar findings. For instance, studies from Europe and North America confirm the significance of GTR in improving survival outcomes in GBM patients [20]. Similarly, studies have highlighted the importance of MGMT methylation in predicting better survival outcomes, with patients showing methylated MGMT promoters experiencing longer overall survival than those with unmethylated MGMT [22,23].

In Pakistan, there is a notable gap in research on the long-term survival of GBM patients, particularly in relation to the surgical approach and molecular markers like MGMT methylation. While a few studies have explored the outcomes of various treatment modalities, comprehensive studies focusing on long-term survival and its molecular predictors, including MGMT, KPS, and surgical resection type, are scarce. This study provides new insights into these crucial factors, marking a significant contribution to the scientific literature from Pakistan. The lack of large cohort studies on this subject in Pakistan highlights the need for further research in this area.

Study limitations and future directions

While this study provides valuable insights into the long-term outcomes of GBM patients, it has a few limitations. The retrospective nature of the study and the limited sample size may introduce biases in the interpretation of the results. Additionally, data on factors such as the genetic profiles beyond MGMT, IDH mutations, and other potential biomarkers were not available, which could have provided a more comprehensive view of the molecular landscape of long-term survivors. Future studies should focus on larger, multicenter cohorts to validate these findings and include additional genetic and epigenetic markers, such as TERT mutations and IDH1/2 mutations, to enhance the understanding of survival predictors in GBM.

This study contributes significantly to the understanding of long-term survival in GBM patients, particularly in the context of Pakistan, and provides a comparison to international research. Future research should aim to expand upon these findings with larger, prospective studies and explore the role of newer biomarkers in predicting GBM outcomes.

## Conclusions

Our approach identifies maximal safe resection, adjuvant therapy optimization, and molecular profiling as critical factors influencing long-term survival in GBM while also revealing their synergistic effects on prognosis. These findings support the adoption of personalized, multimodal treatment strategies to improve outcomes. Future research should focus on refining resection boundaries using advanced intraoperative imaging, developing novel targeted therapies based on genetic subtyping, and exploring immunotherapeutic combinations to overcome treatment resistance, ultimately paving the way for more effective GBM management.
